# Supplementary material for: Resolving the full spectrum of human genome variation using Linked-Reads
Source: Genome Res. 2019 Apr;29(4):635–45. doi: 10.1101/gr.234443.118 (PMC6442396; doi:10.1101/gr.234443.118)
Supplement: Supplemental Material [file supp_gr.234443.118_Supplemental_longranger-2.2.zip › longranger-2.2.tar.bz2!/lr2.2-public/lib/stan/cmdstan/stan/doc/api/html/matrix__vari_8hpp_source.html]

Stan: src/stan/agrad/rev/internal/matrix\_vari.hpp Source File


|  |  |
| --- | --- |
|  | Stan  2.5.0  probability, sampling & optimization |


- Main Page
- Related Pages
- Namespaces
- Classes
- Files

- File List
- File Members

All Classes Namespaces Files Functions Variables Typedefs Enumerations Enumerator Friends Macros Pages

- src
- stan
- agrad
- rev
- internal

matrix\_vari.hpp

Go to the documentation of this file.

1 #ifndef STAN\_\_AGRAD\_\_REV\_\_INTERNAL\_\_MATRIX\_VARI\_HPP

2 #define STAN\_\_AGRAD\_\_REV\_\_INTERNAL\_\_MATRIX\_VARI\_HPP

3

4 #include <stan/math/matrix/Eigen.hpp>

5 #include <stan/agrad/rev/var.hpp>

6 #include <stan/agrad/rev/vari.hpp>

7

8 namespace stan {

9  namespace agrad {

10

11  class op\_matrix\_vari : public vari {

12  protected:

13  const size\_t size\_;

14  vari\*\* vis\_;

15  public:

16  template <int R, int C>

17  op\_matrix\_vari(double f, const Eigen::Matrix<stan::agrad::var,R,C>& vs) :

18  vari(f),

19  size\_(vs.size()) {

20

21  vis\_ = (vari\*\*) operator new(sizeof(vari\*) \* vs.size());

22  for (int i = 0; i < vs.size(); ++i)

23  vis\_[i] = vs(i).vi\_;

24  }

25  vari\* operator[](size\_t n) const {

26  return vis\_[n];

27  }

28  size\_t size() {

29  return size\_;

30  }

31  };

32

33  }

34 }

35 #endif

vari.hpp

stan::agrad::op\_matrix\_vari::operator[]

vari \* operator[](size\_t n) const

**Definition:** matrix\_vari.hpp:25

stan::agrad::vari

The variable implementation base class.

**Definition:** vari.hpp:28

var.hpp

stan::agrad::op\_matrix\_vari::size\_

const size\_t size\_

**Definition:** matrix\_vari.hpp:13

Eigen.hpp

stan::agrad::op\_matrix\_vari::vis\_

vari \*\* vis\_

**Definition:** matrix\_vari.hpp:14

stan::agrad::op\_matrix\_vari::size

size\_t size()

**Definition:** matrix\_vari.hpp:28

stan::agrad::op\_matrix\_vari::op\_matrix\_vari

op\_matrix\_vari(double f, const Eigen::Matrix< stan::agrad::var, R, C > &vs)

**Definition:** matrix\_vari.hpp:17

stan::agrad::op\_matrix\_vari

**Definition:** matrix\_vari.hpp:11


---

[ Stan Home Page ]

*© 2011–2014,
Stan Development Team.*
